# Covid-19 and non-communicable diseases: evidence from a systematic literature review

**DOI:** 10.1186/s12889-021-11116-w

**Published:** 2021-06-05

**Authors:** Zlatko Nikoloski, Ada Mohammed Alqunaibet, Rasha Abdulrahman Alfawaz, Sami Saeed Almudarra, Christopher H. Herbst, Sameh El-Saharty, Reem Alsukait, Abdullah Algwizani

**Affiliations:** 1 London School of Economics and Political Science, London, UK; 2 Public Health Authority, Riyadh, Saudi Arabia; 3 Ministry of Health, Riyadh, Saudi Arabia; 4 Health, Nutrition and Population Global Practice, World Bank Group, Riyadh, Saudi Arabia; 5 Health, Nutrition and Population Global Practice, World Bank Group, Kuwait City, Kuwait

**Keywords:** Non-communicable diseases, Covid-19, Clinical outcomes

## Abstract

**Background:**

Since early 2020, the Covid-19 pandemic has engulfed the world. Amidst the growing number of infections and deaths, there has been an emphasis of patients with non-communicable diseases as they are particularly susceptible to the virus. The objective of this literature review is to systematize the available evidence on the link between non-communicable diseases and Covid-19.

**Methods:**

We have conducted a systematic review of the literature on Covid-19 and non-communicable diseases from December, 2019 until 15th of November, 2020. The search was done in PubMed and in doing so we used a variety of searching terms in order to isolate the final set of papers. At the end of the selection process, 45 papers were selected for inclusion in the literature review.

**Results:**

The results from the review indicate that patients with certain chronic illnesses such as diabetes, hypertension (and other cardiovascular diseases), chronic respiratory illnesses, chronic kidney and liver conditions are more likely to be affected by Covid-19. More importantly, once they do get infected by the virus, patients with chronic illnesses have a much higher likelihood of having worse clinical outcomes (developing a more severe form of the disease or dying) than an average patient. There are two hypothesized channels that explain this strong link between the chronic illnesses enumerated above and Covid 19: (i) increased ACE2 (angiotensin-converting enzyme 2) receptor expressions, which facilitates the entry of the virus into the host body; and (ii) hyperinflammatory response, referred to as “cytokine storm”. Finally, the literature review does not find any evidence that diabetes or hypertension related medications exacerbate the overall Covid-19 condition in chronic illness patients.

**Conclusions:**

Thus, the evidence points out to ‘business as usual’ disease management model, although with greater supervision. However, given the ongoing Covid-19 vulnerabilities among people with NCDs, prioritizing them for the vaccination process should also figure high on the agenda on health authorities.

**Supplementary Information:**

The online version contains supplementary material available at 10.1186/s12889-021-11116-w.

## Introduction

The novel Respiratory Syndrome Coronavirus-2 (SARS-CoV-2) caused a cluster of pneumonia cases in China at the end of 2019. After a few months, it led to a pandemic that has spread throughout most countries of the world. SARS-CoV-2 disease (Covid-19) primarily manifests as a lung infection and its clinical course is characterized by respiratory symptoms ranging from a mild respiratory infection (including fever, cough and fatigue) to pneumonia, acute respiratory distress syndrome (ARDS), shock, and death. While Covid-19 had been initially considered as a respiratory infection, causing harm primarily through inflammatory and immunological processes in the respiratory tract, emerging evidence points out that patients with non-communicable diseases (NCDs) are at higher risk of contracting Covid-19 and suffering worse consequences; moreover, emerging evidence points to a strong feedback mechanism between Covid-19 and existing chronic illnesses (e.g. diabetes) thus further contributing to organ damage and fatal consequences.

The interplay between Covid-19 and NCD shows a set of different effects, both direct and indirect. Direct effects relate primarily to the fact that there is a significant number of preliminary reports connecting certain pre-existing conditions, such as cardiac failure, coronary heart disease, hypertension and diabetes, to a more severe course of Covid-19. Thus, comorbidities may play an important role both in increased susceptibility for infection with SARS-CoV-2 as well as increase the risk of a more severe course of the disease. For now, it seems that an important mechanism is inflammation in the small vessels, particularly in the heart and lungs, but potentially also in other organs, e.g. digestive tract.

Indirect effects are more difficult to measure as they may range from the avoidance of using health services due to the fear of infection. This may lead to: (a) delays in the diagnosis of more acute conditions, such as acute myocardial infarction (AMI) or stroke (CVI); (b) skipping screening appointments or their cancellation due to the running epidemic; (c) lengthening of the waiting lists for diagnostic and therapeutic procedures.

An important feature of the Covid-19 pandemic is also the fact that the knowledge about it is only being gained and it is still unfolding. We are faced with an interesting challenge, where it has become easy to publish quickly about the different findings. But this goes against the scientific rigor in some cases. It is essential to exert an above average level of caution when interpreting results.

Against this background, we conduct this literature review with the main purpose of shedding more light on: (i) the prevalence of Covid-19 (and hence susceptibility) among patients with chronic illnesses; (ii) the analytical link between NCDs, disease progression and disease outcome among patients with selected NCDs; (iii) the pathways through which Covid-19 impacts upon patients with various NCDs from a clinical perspective and (iv) to provide a more definitive answer on the link between medications used to manage various NCDs and Covid-19 progression and outcome.

## Methodology

We conducted literature review on published papers from 31st of December, 2019 until the 15th of November, 2020. The search was done in PubMed and in doing so we used a variety of searching terms in order to isolate the final set of papers. The template below provides a snapshot of the search mechanisms that we used:

(“covid 19” or “COVID 19” or “sars Cov 2” or “coronavirus” or “corona virus”) and (“non communicable diseases” or “non communicable disease” or “NCD” or “NCDs” or “chronic illnesses” or “chronic diseases”)

As well as

(“covid 19” or “COVID 19” or “sars Cov 2” or “coronavirus” or “corona virus”) and (“diabetes” or “cardiovascular diseases” or “hypertension” or “cancer” or “kidney disease”).

The studies that were extracted were carefully examined. The inclusion criteria included, inter alia, prevalence of the NCDs in the search criteria. Moreover, we also included studies that reported clinical outcomes in form of disease severity as well as death. The studies were limited to adult humans and had to be written in English in order to be considered. Duplicate studies, letters, case reports, abstracts, studies written in languages other than English were excluded.

No ethical approval was needed, given that this was a literature review of published studies.

**Fig. 1:**
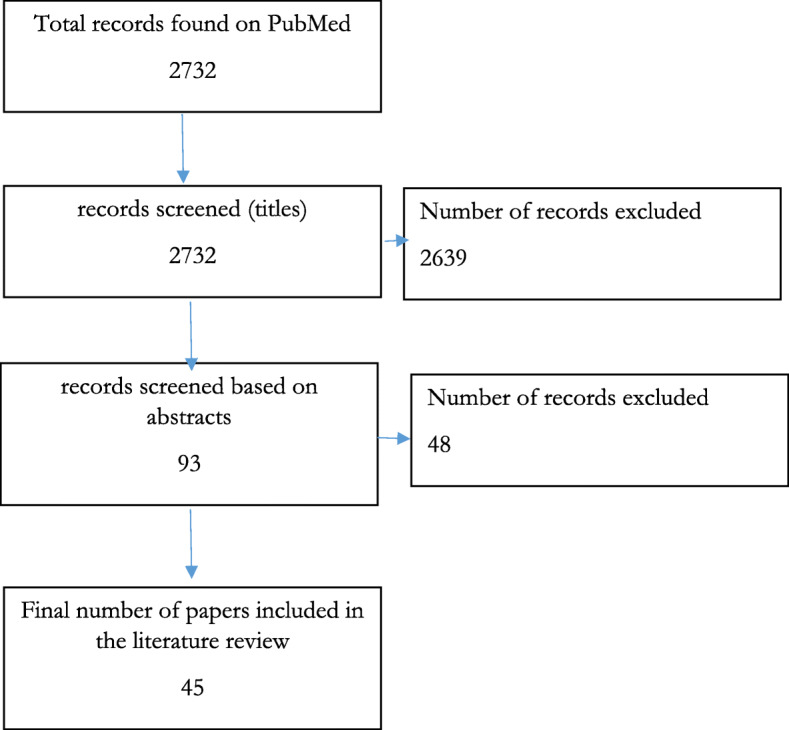
Flow chart of the publication selection process. Source: Authors

Figure 1 above provides a snapshot of the selection criteria for the papers in this literature review. Initially, 2732 records were identified and after screening for titles, abstracts and full papers, 45 records were retained which were used in this literature review and which we elaborate on throughout this paper.

## Results

### Covid-19 and diabetes

Most of the studies included in this literature review study the link between Covid-19 and diabetes (Table 1). A strand of this literature has focused on studying the prevalence of diabetes in Covid-19 patients, albeit descriptively. According to the existing studies, the prevalence of diabetes in Covid-19 patients varies, but it is almost always in double digit levels. More specifically, the prevalence of diabetes among Covid-19 patients ranges from 14% [1], 17% [2], 22% [3] to 44% [4] (Additional file 1: Table A1).

**Table 1:** Type of studies included in the literature review

Chronic illness	Number of studies	Share of total studies included in the review	Type of studies included, (N)
Diabetes	33	73	Retrospective, observational study (2)
			Retrospective, case study (3)
			Literature review (24)
			Meta-analysis (1)
			Clinical study (1)
			Multi-centre cross sectional study (1)
			National reporting data analysis (1)
Hypertension/cardiovascular disease	28	62	Nested case-control design (1)
			Literature review (22)
			Observational study (1)
			Retrospective case series analysis (2)
			Multi-centre cross sectional study (1)
			National reporting data analysis (1)
COPD/other respiratory illnesses	11	24	retrospective cohort study (1)
			clinical analysis of electronic records (1)
			literature review (8)
			Retrospective case series analysis (1)
Chronic kidney disease	9	20	Retrospective case series analysis (1)
			literature review (8)
Cancer	10	22	retrospective cohort study (2)
			Literature review (7)
			Multi-centre cross sectional study (1)
Chronic liver disease	4	9	Literature review (4)
Asthma	7	16	demographic analysis (1) Study of medical records (1)
			Literature review (5)

A special strand of the literature has also focused on a more analytical link between diabetes and Covid-19, focusing on a few specific outcomes, such as mortality or severity of the disease. The common thread in this strand of the literature is that patients with diabetes show a consistently lower likelihood of survival or recovery and are much more likely to have a severe disease progression, compared to the non-diabetic patients.

In a retrospective case series, Yan et al. [5] for example, report that the survival rate was lower among the diabetic patients compared to the non-diabetic ones. More specifically, in their study, the HR was 1.5 (95% CI 1.0–2.3) after adjustment for demographic factors. Similarly, Yan et [6] find the patients with diabetes had consistently and independently poorer outcomes with a relative risk of dying at 3.0 (95% CI 1.3–6.8). In the context of Mexico, a study also found that diabetes is associated with hospitalization and worse outcomes among patients with Covid-19. These findings are echoed in some existing literature reviews [7]. For example, in a study by Du et al. [8], the risk of severe cases was higher in Covid-19 patients with diabetes (RR = 2.1, 95%CI 1.8–2.6) and the risk of death was also higher in Covid-19 patients with diabetes (RR = 3.2, 95%CI 2.6–3.8). Furthermore, and going beyond mortality as an outcome, Praveen et al. [9] find that diabetes was lower in the survivors (OR: 0.6; 95%CI: 0.4–0.9) and non-severe patients (OR: 1.7; 95%CI 1.2–2.3). Finally, Noor et al. [10] found a significant association between Covid-19 and mortality among diabetes patients (RR 1.9, 95% CI 1.2–2.8).

A few of the literature reviews that we include have conducted meta-analyses in order to further unearth the link between diabetes and Covid-19 mortality. In most of the cases, the authors find statistically significant link between diabetes and dying from Covid-19 with odds ratios higher than one. In a meta-analysis by Wu et al. [11], the authors find a close link between diabetes and mortality with OR of 1.75. In another literature review conducted by Ssentonoga et al. [12], diabetes was associated with a significantly greater risk of mortality from Covid-19 (OR 1.5, 95% CI 1.0–2.2). In the rest of the studies, the odds ratios capturing the link between diabetes and Covid-19 mortality teeter around 2.5 [13–17]. In only one of the studies the odds ratios of death due to Covid-19 among diabetics were higher than 3. More specifically, Lu et al. [18] find that diabetes comorbidity was one of the key mortality risk factors (OR = 3.7, 95% CI 2.4–5.9). Furthermore, and taking a more comprehensive approach Awortwe et al. [19] suggest that cardio-metabolic syndrome (mainly characterized by insulin resistance, impaired glucose tolerance, dyslipidemia, hypertension, and central adiposity) is associated with negative clinical outcomes including mortality (risk difference RD 0.1, 95%-CI 0.1–0.2), admission to ICU (RD 0.1, 95%-CI 0.04–0.2) and severe infection (RD 0.1, 95%-CI 0.01–0.09) in Covid-19 patients (Additional file 1: Table A1).

### Covid-19 and hypertension/cardiovascular diseases

The second most studied set of chronic illnesses are cardiovascular illnesses, including hypertension (Table 1). Similarly, to the case of diabetes, one strand of the literature has focused descriptively on the prevalence of hypertension and cardiovascular diseases among Covid-19 patients. In a literature review, Wolff et al. [20], establish that, along with diabetes, hypertension and other cardiovascular diseases are the most prevalent chronic illnesses among Covid-19 patients. The prevalence of hypertension in Covid-19 patients vary, from 15.6% [4], 16.4% [21], 22% [1], 27.4% [2] to 38.6% [3]. Similarly, the prevalence of other cardiovascular diseases is in double-digits varying form 4.7% [4], 8.9% [2], 12.1% [21], 13% [1] to 17.5% [3]. (Additional file 1: Table A2).

In the second strand of the literature, the authors establish a more analytical link between hypertension (and the rest of the cardiovascular diseases) and negative clinical outcomes (e.g. death or severity of illness) among Covid-19 patients. In that respect, the link between hypertension and Covid-19 outcomes is particularly studied. A very comprehensive literature review by Pranata et al. [22] suggests that hypertension was associated with increased composite poor outcome (risk ratio (RR) 2.1 95% CI 1.9–2.4) and its sub-group, including mortality (RR 2.2 95% CI 1.7–2.8), severe Covid-19 (RR 2.0, CI 1.7–2.5), ARDS (RR 1.6, 95% CI: 1.1–2.4), ICU care (RR 2.1, 95% CI: 1.3–3.3), and disease progression (RR 3.0, 95% CI: 1.5–6.0). Similarly, in a literature review by Parveen et al. [9], hypertension was positively associated with death (OR: 0.5; 95% CI: 0.3–0.7), ICU care (OR: 0.4; 95%CI: 0.2–0.8) and severity (OR: 2.7; 95% CI 1.3–5.7) (Additional file 1: Table A2).

While establishing a link between hypertension and Covid-19 mortality, most of the literature reviews also conduct meta-analyses, which find significantly higher odds of Covid-19 mortality among hypertensive patients, with odds ratios usually ranging from 2.5 to 3 [10, 12–14, 23–25]. Finally, only in one of the meta-analyses were the odds ratios of dying from Covid-19 among hypertensive patients was higher than 3. More specifically, a literature review by Liu et al. [17] confirms the finding that hypertension (OR 3.4, 95% CI 2.5–4.7) was one of the key mortality risk factors.

Similarly, to hypertension, a strand of this literature has focused exclusively on the clinical outcomes of Covid-19 patients with other cardio-vascular comorbidities. In a study by Gu et al. [26], the estimated mortality risk in patients with pre-existing coronary heart disease (CHD) was three times that of those without CHD. The estimated 30-day survival probability for a profile patient with pre-existing CHD (65-year-old woman with no other comorbidities) was 0.5 (95% CI 0.3–0.8). Furthermore, a study in Oman [27] found that patients with cardiac injury had higher mortality than those without cardiac injury (53.3% vs 7.1%). The literature review by de Almeida-Pittito [13] mentioned above also suggested that cardiovascular disease was strongly associated with both severity and mortality, respectively (OR 4.0 95% CI 2.8–5.9 and OR 6.3 95% CI 3.7–10.8) also reflecting the previous findings [10]. In addition, the literature review by Matshushita et al. [23] suggested that acute myocardial injury, determined by elevated high-sensitivity troponin levels, is commonly observed in severe cases, and is strongly associated with mortality. Moreover, a comprehensive review by Ssentonoga et al. [12] found that cardiovascular disease (risk ratio (RR) 2.3, 95% CI 1.6–3.2) and congestive heart failure (RR 2.03 95% CI 1.3–3.2) were associated with a significantly greater risk of mortality from Covid-19. A review by Khan et al. [16] found that higher likelihood of dying was found among Covid-19 patients who had pre-existing cardiovascular diseases (odds ratio 3.4 95% CI 2.9–4.1), reflecting the findings by Liu et al. [17]. Finally, a literature review by Hessami et al. [28] indicated that acute cardiac injury, (OR: 13.3, 95% CI 7.4–24.0), heart failure (OR: 6.7, 95% CI 3.3–13.5), arrhythmia (OR: 2.8, 95% CI 1.4–5.3), coronary artery disease (OR: 3.8, 95% CI 2.4–5.9), and cardiovascular disease (OR: 2.6, 95% CI 1.9–3.6) were significantly associated with Covid-19 mortality (Additional file 1: Table A2).

### Covid-19 and COPD

The third most prevalent chronic illness associated with negative outcomes due to Covid-19 is COPD (chronic obstructive pulmonary disease) as well as other underlying chronic illnesses. As in the rest of the literature, here as well, there are two strands that emerge: one of them is focused on the link between Covid-19 and chronic respiratory illnesses from a descriptive point of view, while the second strand is more analytical. In their literature review, Mahmud et al. [1] and Bajgain et al. [2] find that most prevalent chronic comorbid conditions were, inter alia, respiratory diseases (5%).

The second strand of the literature is more analytical and tries to establish a more robust link between pre-existing chronic lung illnesses and Covid-19 disease outcomes. Nachtigall et al. [29] for example argue that pre-existing lung disease was one of the main predictors of death (HR 1.6; 95%CI 1.2–2.2). Similarly, Lu et al. [18] in a literature review suggest that chronic lung disease (OR 3.4, 95% CI 1.8–6.5) was one of the key mortality risk factors, which is further echoed in the other studies included in this literature review [14, 16, 17] (Additional file 1: Table A3).

Finally, a special strand of the literature has focused on COPD as the most dominant pulmonary chronic illness and its link with Covid-19. Graziani et al. [30] find that compared with COPD-free individuals, COPD patients with Covid-19 showed significantly poorer disease prognosis, as evaluated by hospitalizations (31.1% vs. 39.8%: OR 1.6; 95% CI 1.1–1.2) and mortality (3.4% vs. 9.3%: OR 2.9; 95% CI 2.3–3.8). In their literature review, Awortwe et al. [19], indicated that chronic obstructive pulmonary disease, inter alia, worsen the clinical outcomes including mortality (risk difference RD 0.1, 95%-CI 0.05–0.2), admission to ICU (RD 0.1, 95%-CI 0.04–0.2) and severe infection (RD 0.05, 95%-CI 0.01–0.09) in Covid-19 patients (Additional file 1: Table A3).

### Covid-19 and chronic kidney disease

The fourth most common chronic illnesses associated with negative outcomes related to Covid-19 are chronic kidney diseases. In a literature review by Bajgain et al. [2], around 2.6% of patients with chronic kidney disease also had Covid-19.

A literature review by Awowrtwe et al. [19] found a significantly higher likelihood of poor Covid-19 outcomes among patients with chronic kidney disease. Results suggested that chronic kidney disease, inter alia, was associated with worse clinical outcomes including mortality (risk difference RD 0.1, 95%-CI 0.1–0.12), admission to ICU (RD 0.1, 95%-CI 0.04–0.2) and severe infection (RD 0.05, 95%-CI 0.01–0.09) in Covid-19 patients. A literature review by Sepandi et al. [14] found that some chronic diseases such as kidney disorder (OR 2.6 95% CI 1.2–5.6) can increase the risk of Covid-19 mortality, which is similar to the rest of the studied included in this review [12, 16, 31] (Additional file 1: Table A4).

### Covid-19 and cancer

As in the rest of the chronic illnesses the literature on the link between Covid-19 and cancers could be divided into two strands. In the first one, authors are mainly concerned with finding the prevalence of cancer among Covid-19 patients. The literature reviews that we cover suggest prevalence of cancer among Covid-19 patients ranging from 1.2% [4] to 3.5% [2] and 8% [1] (Table A5).

The second strand of the literature has analytically established a link between cancer and Covid-19 outcomes. In a literature review by Noor et al. [10] a significant association were found between mortality among Covid-19 infected patients and cancer (RR 2.3, 95% CI 1.8–3.0). Similarly, a literature review by Ssentonoga et al. [12] found that cancer (1.5 95% CI 1.01 to 2.2) was associated with a significantly greater risk of mortality from Covid-19. In their own review, Khan et al. [16] suggest higher likelihood of deaths was found among Covid-19 patients who had any types of cancers (OR 2.2, 95% CI 1.6–3.0). Finally, and specifically focusing on cancer patients, Zhang et al. [32] find a significantly higher mortality rate, particularly if the anti-tumour treatment was within the last 14 days. There is a nuance however. While existing evidence finds that the fatality rate in the lung cancer patients with Covid-19 was 32.9% (95% CI 27.9 to 38.0%) and the fatality rate in haematological cancer patients was 34.2% (95% CI 23.1 to 46.2%), in other types of solid cancer excluding lung, the overall case fatality and severe event rates were 17.2% (95% CI 12.3 to 22.7%) [33]. Similarly, another study finds that patients with solid versus hematologic cancers exhibit different clinical outcomes, with patients with hematologic cancers having a significantly higher mortality relative to patients with solid cancers after accounting for confounders [34].

### Covid-19 and liver disease

The literature on the link between Covid-19 and chronic liver disease is less sanguine. In three of the four studies that we had identified, there is a clear link between existing liver chronic illness and higher likelihood of mortality. Oyelade et al. [35] found that in patients with Covid-19 and underlying liver diseases, 57.3% (43/75) of cases were severe, with 17.65% mortality. Khan et al. [16] found that there was a higher likelihood of deaths was found among Covid-19 patients who had pre-existing liver diseases (OR = 2.4, 95% CI 1.5–3.7), echoing previously established notion [10]. However, in another literature review, Wang et al. [31] found no relationship between Covid-19 mortality and pre-existing liver disease (Table A6).

### Covid-19 and asthma

As in the rest of the cases, a strand of the literature has focused on estimating the prevalence of asthma among Covid-19 patients. In a study in South Korea, the prevalence of asthma among Covid-19 patients was 2.9% [36], somewhat similar to another review which finds that asthma is a pre-morbid condition in about 1.6% of the Covid-19 patients [37]. Another systematic review of the link between asthma and Covid-19 finds a somewhat higher prevalence of asthma among Covid-19 patients (7.46%) [38] echoing the heterogeneity of prevalence across different countries and regions as reported in another systematic review [39].

The second strand of the literature has focused on studying the clinical outcomes of asthma patients with Covid-19. A systematic literature review finds that there was no significant difference in the combined risk of requiring admission to ICU and/or receiving mechanical ventilation for people with asthma (RR 0.87, 95% CI 0.94–1.37) and risk of death from Covid-19 (RR 0.87; 95% CI 0.68–1.10) [38].These findings are similar to the ones conducted in another meta-analysis [40, 41]. Overall, the literature suggests that asthma is not an independent risk factors for the clinical outcomes of Covid-19 [36]. In a study by Chibba et al. [42], asthma was not associated with an increased risk of hospitalization (relative risk, 0.96; 95% CI, 0.8–1.2) after adjusting for age, sex, and comorbidities. Similarly, a literature review by Morais-Almeida et al. [43] found that there is no strong evidence supporting that patients with asthma have a higher risk of becoming seriously ill from coronavirus disease 2019 (Table A7).

## Discussion

There are a few findings that stem from this review on the link between Covid-19 and non-communicable diseases. First, as evidenced by this review, studies have observed a high prevalence of certain chronic illnesses (diabetes, hypertension) among Covid-19 patients. Second, and going beyond descriptive observation, majority of the studies find that Covid-19 patients have higher likelihood of worse clinical outcomes (e.g. higher mortality) compared to patients without chronic illnesses. This is particularly the case for diabetes, hypertension, COPD and chronic kidney disease. Third, while our findings are similar for the rest of the chronic illnesses featured in this review, they are less sanguine in the case of chronic liver disease. Finally, the result of the literature review suggests no link between asthma and Covid-19.

While the research on the interplay between diabetes and Covid-19 is still ongoing, there are a few preliminary findings/research hypotheses that have been put forth as to why diabetic patients are associated with more pronounced Covid-19 complications.

First, the existing knowledge suggests that patients with chronic illnesses (diabetes, hypertension, other cardio-vascular diseases, chronic kidney disease) have increased ACE2 (angiotensin-converting enzyme 2) receptor expressions, which facilitates the entry of the virus into the host body [44]. Moreover, as the study by Erener et al. [45] suggests, ACE2 is expressed in various tissues including the lung, heart, kidney tubules, the luminal surface of the small intestine, blood vessels, endocrine and exocrine pancreas [45]. Similar explanations, specific to cardio-vascular diseases have been put forth by Pranata et al. [22]. In the case of asthma, it has been argued that respiratory epithelial cells in patients with asthma have decreased gene expression for ACE2 receptors and therefore may be protective against Covid-19 infection [46].

Another potential reason for the increased risk of severe Covid-19 disease in patients with chronic illnesses might be attributed to the hyperinflammatory response, referred to as “cytokine storm” [47, 44]. Patients with certain chronic illnesses (e.g. diabetes, hypertension) suffer from a continuous low-grade inflammation facilitating the emergence of a cytokine storm, which in turn appears to be directly related to the severity of Covid-19 pneumonia cases and to subsequent death [47]. More specifically, patients with diabetes appear to have an impaired adaptive immune response characterized by an initial delay of Th1 cell-mediated immunity and a late hyperinflammatory response. In the absence of an immunostimulant, diabetes is associated with an increased pro-inflammatory cytokine response marked by increased secretion of IL-1, IL-6, IL-8 and TNF-α, which in turn play a more deleterious role in Covid-19 infection [44, 45]. When specifically focusing on cancer, ACE2 and TMPRSS2 expression is found higher in cancer patients, and coagulopathy is a potential risk observed in a number of cancer patients [48]. Different hypotheses have been put forth regarding the differences in clinical outcomes between Covid-19 patients with solid vs. hematologic cancers. A study has found that the principal cause of elevated mortality risk from Covid-19 in solid cancer patients is cancer progression [49]. In contrast, the same study suggests that in haematological cancer patients, there was a particularly striking expression of exhaustion markers by CD8+ T cells. Exhausted T cells, in turn, may compromise virus clearance [49].

These links between Covid-19 and some of the chronic illnesses mentioned above have implications on the impact of current medical treatments for certain chronic illnesses on the probability of developing severe Covid-19. However, the results presented in studies covered in this literature review reveal that there is no evidence to support this hypothesis currently. In view of lack of robust evidence for either benefit or harm, it is reasonable for patients to continue using ACE inhibitors and ARB, as recommended by European Society of Cardiology Council on Hypertension, European Society of Hypertension and American Heart Association [44]. Moreover, there are several studies about the protective effect of statins in pneumonia [44]. Statins are known to increase ACE-2 levels and may protect against viral entry of SARS CoV-2. However, this increase in ACE-2 could be counterintuitive in the current context. Nevertheless, statins are known to inhibit Nuclear factor kappa B (NFkB) activation and might help in blunting the cytokine storm [44].

Similarly, to the case of diabetes, the literature review on the link between medications prescribed for managing hypertension and severity of Covid-19 finds no conclusive evidence. In a review by Hessami et al. [28] in 9 studies that were included, with a total of 10.900 Covid-19 cases, the random-effects analysis showed a combined OR for severity 0.76 (95% CI 0.39–1.49). Moreover, in their study there was a high heterogeneity indicating that there was no association between use of ACEI/ARB and Covid-19 severity. Continuation of ongoing treatment, coupled with self-management and remote interventions has also been suggested in the context of other chronic illnesses such as asthma [50].

## Conclusions and policy implications

There are a few conclusions that stem from this comprehensive literature review on the link between NCDs and Covid-19. First, patients with certain chronic illnesses such as diabetes, hypertension (and other cardiovascular diseases), chronic respiratory illnesses, chronic kidney and liver conditions are more likely to be affected by Covid-19. This is further attested by the high prevalence of some of these chronic illnesses (such as diabetes and hypertension) among Covid-19 patients. More importantly, once they do get infected by the virus, patients with chronic illnesses have a much higher likelihood to either develop a more severe illness than an average patient; moreover they are more likely to die relative to patients who do not have chronic illnesses. Our literature review presents evidence on this obtained both from case- controlled studies as well as from other literature reviews that we distilled. Third, while the research on the reasons behind the high susceptibility of NCD patients to Covid-19 is still ongoing, researchers have hypothesised two main channels: (i) increased ACE2 (angiotensin-converting enzyme 2) receptor expressions, which facilitates the entry of the virus into the host body; and (ii) hyperinflammatory response, referred to as “cytokine storm”. Our literature review points out that these transmission mechanisms are at play when it comes to all Covid-19/NCD linkages. Finally, the literature review does not find any evidence that diabetes or hypertension related medications exacerbate the overall Covid-19 condition in chronic illness patients. Based on this there are a few implications/policy recommendations that stem from this research. First, it is recommended that patients continued with existing treatment for chronic illnesses, especially as there is no evidence that certain medications (e.g. for managing diabetes or hypertension) are associated with worse Covid-19 clinical outcomes. Second, there should be a greater emphasis on telemedicine and virtual visits. With physical distances no longer a factor, virtualizing the care provided by diabetes educators, dieticians, and specialized mental health professionals could improve access further than what was previously possible with in-person encounters [51]. Third, through the virtual visits there should also be an improvement in patient education and self-management [52]. Fourth, given the ongoing Covid-19 vulnerabilities among people with NCDs, prioritizing them for the vaccination process should also figure high on the agenda on health authorities. Finally, all of the steps above have to be done in resource-constrained setting, with resources being diverted to Covid-19 needs. Thus and going beyond just covering the immediate needs to patients, health system strengthening, while putting particular emphasis on primary healthcare, could go long way in providing effective and safe management of chronic illnesses [53].

## Supplementary Information

**Additional file 1: Table A1.** Covid-19 and diabetes: overview of the papers included in this literature review. **Table A2.** Covid-19, hypertension and cardiovascular diseases: overview of the papers included in this literature review. **Table A3.** Covid-19, COPD and other chronic respiratory illnesses: overview of the papers included in this literature review. **Table A4.** Covid-19 and chronic kidney disease: overview of the papers included in this literature review. **Table A5.** Covid-19 and cancer: overview of the papers included in this literature review. **Table A6.** Covid-19 and chronic liver disease: overview of the papers included in this literature review. **Table A7.** Covid-19 and asthma: overview of the papers included in this literature review.

## Data Availability

Not applicable.

## Abbreviations

ARDS: Acute respiratory distress syndrome
NCD: Noncommunicable disease
AMI: Acute myocardial infarction
CI: Confidence interval
OR: Odds ratios
ICU: Intensive care unit
CHD: Coronary heart disease
COPD: Chronic obstructive pulmonary disease
ACE2: Angiotensin-converting enzyme 2
