# Supplementary material for: Covid-19 and non-communicable diseases: evidence from a systematic literature review
Source: BMC Public Health. 2021 Jun 5;21:1068. doi: 10.1186/s12889-021-11116-w (PMC8178653; doi:10.1186/s12889-021-11116-w)
Supplement: Supplementary file 1 — Additional file 1: Table A1. Covid-19 and diabetes: overview of the papers included in this literature review. Table A2. Covid-19, hypertension and cardiovascular diseases: overview of the papers included in this literature review. Table A3. Covid-19, COPD and other chronic respiratory illnesses: overview of the papers included in this literature review. Table A4. Covid-19 and chronic kidney disease: overview of the papers included in this literature review. Table A5. Covid-19 and cancer: overview of the papers included in this literature review. Table A6. Covid-19 and chronic liver disease: overview of the papers included in this literature review. Table A7. Covid-19 and asthma: overview of the papers included in this literature review. [file 12889_2021_11116_MOESM1_ESM.docx]

Table A1. Covid-19 and diabetes: overview of the papers included in this literature review

| Author, year | Study type | Number of patients | Location | Main findings |
| --- | --- | --- | --- | --- |
| Yan et al, 2020a | single-centre, retrospective, observational study | 193 | Wuhan, China | survival rate lower among diabetes patients. The HR was 1.53 (95% CI 1.02 to 2.30; p=0.041) after adjustment for demographic factors. |
| Yan et al, 2020b | retrospective case series | 218 | Loudi, Shaoyang, and Xiangtan, China | The presence of diabetes (relative risk (RR)), 3.0; 95% CI, 1.3–6.8; p = 0.007) independently associated with poor outcome. |
| Wolff et al, 2020 | literature review | n/a | n/a | The most common pre-existing comorbidity is, inter alia, diabetes. |
| Praveen et al, 2020 | literature review | n/a | n/a | Diabetes was lower in the survivors (OR: 0.56; 95%CI: 0.35–0.90; p = 0.017; I2: 0.0%) and non-severe (OR: 1.66; 95%CI: 1.20–2.30; p = 0.002; I2: 0.0%) patients. No association of diabetes was found with ICU care. |
| Wang et al, 2020 | retrospective study | 605 | Wuhan, China | FBG ≥7.0 mmol/l (HR 2.30 [95% CI 1.49, 3.55]) was independent predictor for 28-day mortality. The OR for 28-day in-hospital complications in those with FBG ≥7.0 mmol/l and 6.1–6.9 mmol/l vs <6.1 mmol/l was 3.99 (95% CI 2.71, 5.88) or 2.61 (95% CI 1.64, 4.41), respectively. |
| Wu et al, 2020 | meta analysis | 926 | n/a | close relationship between diabetes and mortality of Covid-19, with a pooled OR of 1.75 (95% CI 1.31–2.36; P = 0.0002). |
| Du et al, 2020 | literature review | 49564 | n/a | The risk of severe cases (RR = 2.13, 95%CI: 1.76-2.56, I2 = 49%) and the risk of death was also higher in COVID- 19 patients with diabetes (RR = 3.16, 95%CI: 2.64-3.78, I2 = 34%). |
| Chen et al, 2020 | clinical study | 208 | China | In Covid-19 patients, there is a higher prevalence of diabetes and other chronic illnesses. |
| Sanyaolou et al, 2020 | literature review | n/a | n/a | From what is known at the moment, patients with Covid-19 disease who have comorbidities, such as hypertension or diabetes mellitus, are more likely to develop a more severe course and progression of the disease. |
| Lu et al, 2020 | literature review | 16095 | n/a | diabetes comorbidity (OR = 3.73, 95% CI 2.35–5.90) was one of the key mortality risk factors. |
| Giannouchos et al, 2020 | retrospective case series analysis | 236,439 | Mexico | Diabetes was one of the key factors associated with hospitalization and adverse outcome. |
| Awortwe et al, 2020 | literature review | 5586 | n/a | cardiometabolic syndrome (e.g. cardiovascular disease, cerebrovascular disease, hypertension, and diabetes) associated with worsen the clinical outcomes including mortality (risk difference RD 0.12, 95 %-CI 0.05􀀀 0.19, p =0.001), admission to ICU (RD 0.10, 95 %-CI 0.04􀀀 0.16, p = 0.001) and severe infection (RD 0.05, 95 %-CI 0.01􀀀 0.09, p = 0.01) in Covid-19 patients. |
| Singh et al, 2020 | literature review | 7162 | n/a | There is evidence of increased incidence and severity of Covid-19 in patients with diabetes. Covid-19 could have effect on the pathophysiology of diabetes. |
| de Almeida‑Pititto et al, 2020 | literature review | 4305 | n/a | The random-effect meta-analysis showed that diabetes mellitus was associated with severity and mortality [OR 2.35 95% CI 1.80–3.06 and OR 2.50 95% CI 1.74–3.59] |
| Noor et al, 2020 | literature review | 122,191 | n/a | Significant association were found between mortality among Covid-19 infected patients and diabetes [RR 1.87, 95% CI (1.23–2.84), p < 0.001]. |
| Mahumud et al, 2020 | literature review | 202,005 | n/a | Diabetes (14%; 95% CI 12–17%) was one of the most prevalent chronic illnesses among Covid-19 patients |
| Sepandi et al, 2020 | literature review | 944 | n/a | comorbidity with some chronic diseases such as Diabetes type2 OR: 2.42(1.06-5.52)can increase the risk of COVID19 mortality. |
| Qiu et al, 2020 | literature review | 2401 | n/a | The incidence of diabetes among Covid-19 patients was 22.2% (95% CI 19.30 ~ 25.10%) |
| Hu et al, 2020 | literature review | 47,344 | n/a | The prevalence of diabetes was 7.7 % (95CI 6.1–9.3 %) among Covid-19 patients |
| Matsushita et al, 2020 | literature review | 76,638 | n/a | In univariate analyses, factors robustly associated with severe Covid-19 were, inter alia, diabetes (9 studies; 3.20 [2.26–4.53]). |
| Ssentonoga et al, 2020 | literature review | 65,484 | n/a | Diabetes (1.48 [1.02 to 2.15], n = 16) was associated with a significantly greater risk of mortality from Covid-19. |
| Khan et al, 2020 | literature review | 27,670 | n/a | The higher likelihood of deaths was found among Covid-19 patients who had pre-existing immune and metabolic disorders (OR = 2.46, 95% CI = 2.03-2.85). |
| Emami et al, 2020 | literature review | 76993 | n/a | According to the meta-analysis, the pooled prevalence of diabetes among Covid-19 patients was estimated to be 7.87% (95%CI 6.57%-9.28%). |
| Liu et al, 2020 | literature review | 10,948 | n/a | Covid-19 patients who present with either diabetes have a higher likelihood of developing a severe disease with an OR of 2.61 (95% CI 1.93 to 3.52). |
| Lisco et al, 2020 | literature review | n/a | n/a |  |
| Zali et al, 2020 | multi centre cross sectional study | 16035 | Iran | The highest CFR was reported for patients who had diabetes and cardiovascular diseases (38.46%) as underlying non-communicable diseases (NCDs). |
| Gutierrez et al, 2020 | national reporting data analysis | 1,378,002 | Mexico | The study found that obesity, diabetes, and hypertension are positively associated with the three outcomes in a synergistic manner. |
| Erener, 2020 | literature review | n/a | n/a | Based on the new clinical data obtained from Covid-19 patients, a discussion of mechanisms, such as cytokine storm, pulmonary and endothelial dysfunction, and hypercoagulation, that may render individuals with diabetes more vulnerable to Covid-19 is provided. |
| Azar et al, 2020 | literature review | n/a | n/a | The worsened prognosis of Covid-19 patients with diabetes can be attributed to a facilitated viral uptake assisted by the host’s receptor angiotensin-converting enzyme 2 (ACE2). It can also be associated with a higher basal level of pro-inflammatory cytokinespresent in patients with diabetes, which enables a hyperinflammatory “cytokine storm” in response to the virus. |
| Bajgain et al, 2020 | literature review | 22753 | n/a | Major comorbidities seen in patients with Covid-19 were, inter alia, diabetes (17.4%). |
| Flaherty et al, 2020 | literature review | 17845 | n/a | Putative mechanisms of increased Covid-19 disease severity in diabetes include hyperglycaemia, altered immune function, sub-optimal glycaemic control during hospitalisation, a pro-thrombotic and pro-inflammatory state. |
| Wang et al, 2020 | literature review | 6263 | n/a | Pre-existing chronic conditions such as diabetes are strongly associated with an increased risk of developing severe Covid-19. |
| Akbariqomi et al | retrospective, single centre study | 595 | Iran | Significantly, patients with diabetes had more complications and needed more respiratory support than those without diabetes (P < 0.001). At the end of the follow-up, treatment failure and death was significantly higher in patients with diabetes compared to those without diabetes (17.8% vs. 8.7%; P = 0.003). |

Table A2. Covid-19, hypertension and cardiovascular diseases: overview of the papers included in this literature review

| Author, year | Study type | Number of patients | Location | Main findings |
| --- | --- | --- | --- | --- |
| Gu et al, 2020 | nested case–control design | 94 | Hubei Province, China | The estimated mortality risk in patients with pre-existing coronary heart disease (CHD) was three times that of those without CHD (p<0.001). The estimated 30-day survival probability for a profile patient with pre-existing CHD (65-year-old woman with no other comorbidities) was 0.53 (95% CI 0.34 to 0.82). |
| Wolff et al, 2020 | literature review | n/a | n/a | The most common pre-existing comorbidities are, inter alia, hypertension followed by cardiovascular disease. |
| Parveen et al, 2020 | literature review | 2018 | n/a | Hypertension was positively associated with death (OR: 0.49; 95%CI: 0.34–0.73; p<0.001; I2: 0.0%), ICU care (OR: 0.42; 95%CI: 0.22–0.81; p = 0.009; I2: 0.0%) and severity (OR: 2.69; 95%CI: 1.27–5.73; p = 0.01; I2: 52.4%). |
| Feng et al, 2020 | single centre, prospective and observational study | 114 | China | In addition, univariate and multivariate Cox analyses indicated that uncontrolled inflammation responses as well as liver, kidney, and cardiac dysfunction are related to the development of a poor outcome. |
| Sanyaolou et al, 2020 | literature review | n/a | n/a | From what is known at the moment, patients with Covid-19 disease who have comorbidities, such as hypertension, are more likely to develop a more severe course and progression of the disease. |
| Lu et al, 2020 | literature review | 16095 | n/a | hypertension (OR = 3.38, 95% CI 2.45–4.67) was one of the key mortality risk factors. |
| Pranata et al, 2020 | literature review | 6560 | n/a | Hypertension was associated with increased composite poor outcome (risk ratio (RR) 2.11 (95% confidence interval (CI) 1.85, 2.40), p < 0.001; I2, 44%) and its sub-group, including mortality (RR 2.21 (1.74, 2.81), p < 0.001; I2, 66%), severe Covid-19 (RR 2.04 (1.69, 2.47), p < 0.001; I2 31%), ARDS (RR 1.64 (1.11, 2.43), p = 0.01; I2,0%, p = 0.35), ICU care (RR 2.11 (1.34, 3.33), p = 0.001; I2 18%, p = 0.30), and disease progression (RR 3.01 (1.51, 5.99), p = 0.002; I2 0%, p = 0.55). |
| Giannouchos et al, 2020 | retrospective case series analysis | 236,439 | Mexico | Male gender, older age, having one or more comorbidities, such as hypertension were associated with hospitalization and adverse outcome. |
| Al-Wahaibi et al, 2020 | retrospective case series analysis | 143 | Muscat, Oman | Noticeably, patients with cardiac injury had higher mortality than those without cardiac injury (53.3% vs 7.1%; P < 0.00001). |
| Awortwe et al, 2020 | literature review | 5586 | n/a | Results indicated that cardiometabolic syndrome (e.g. cardiovascular disease, cerebrovascular disease, hypertension, and diabetes) were associated with worsen the clinical outcomes including mortality (risk difference RD 0.12, 95 %-CI 0.05􀀀 0.19, p =0.001), admission to ICU (RD 0.10, 95 %-CI 0.04􀀀 0.16, p = 0.001) and severe infection (RD 0.05, 95 %-CI 0.01􀀀 0.09, p = 0.01) in Covid-19 patients. |
| de Almeida‑Pititto et al, 2020 | literature review | 4305 | n/a | The random-effect meta-analysis showed that hypertension was moderately associated respectively with severity and mortality for Covid-19 [OR 2.98 95% CI 2.37–3.75 and OR 2.88 (2.22–3.74)], respectively. Cardiovascular disease was strongly associated with both severity and mortality, respectively [OR 4.02 (2.76–5.86) and OR 6.34 (3.71–10.84)]. |
| Noor et al, 2020 | literature review | 122,191 | n/a | An significant association were found between mortality among Covid-19 infected patients and ,hypertension [RR 2.08,95% CI (1.79–2.43) p < 0.001], cardiovascular disease [RR 2.51, 95% CI (1.20–5.26), p < 0.05]. In addition, significant association for high risk of mortality were also found for coronary heart disease. |
| Matsushita et al, 2020 | literature review | n/a | n/a | Notably, acute myocardial injury, determined by elevated high-sensitivity troponin levels, is commonly observed in severe cases, and is strongly associated with mortality. |
| Mahumud et al, 2020 | literature review | 202,005 | n/a | The most prevalent chronic comorbid conditions were hypertension (22%; 95% CI 17–27%), cardiovascular diseases (13%; 95% CI 10–16%). |
| Sepandi et al, 2020 | literature review | 944 | n/a | The Comorbidity with some chronic diseases such as Hypertension OR: 2.54(1.21-5.32), and Heart diseases OR: 4.37 (1.13-16.90) can increase the risk of COVID19 mortality. |
| Qiu et al, 2020 | literature review | 2401 | n/a | The incidence of hypertension, chronic cardiovascular disease, among the Covid-19 deceased were 38.56% (95% confidence interval (CI) 25.84 ~ 52.12%) and 17.54% (95% CI 13.38 ~ 21.69%), respectively. |
| Hu et al, 2020 | literature review | 47,344 | n/a | The prevalence of hypertension was 15.6 % (95CI 12.6–18.6 %) while cardiovascular disease was 4.7 % (95CI 3.1–6.2 %) among Covid-19 patients |
| Matsushita et al, 2020 | literature review | 76,638 | n/a | In univariate analyses, factors robustly associated with severe Covid-19 were hypertension (8 studies; 2.87 [2.09–3.93]) and CVD (10 studies; 4.97 [3.76–6.58]). |
| Ssentonoga et al, 2020 | literature review | 65,484 | n/a | Cardiovascular disease (risk ratio (RR) 2.25, 95% CI = 1.60–3.17, number of studies (n) =14), hypertension (1.82 [1.43 to 2.32], n = 13), congestive heart failure (2.03 [1.28 to 3.21], n = 3) were associated with a significantly greater risk of mortality from Covid-19. |
| Khan et al, 2020 | literature review | 27,670 | n/a | The higher likelihood of deaths was found among Covid-19 patients who had pre-existing cardiovascular diseases (odds ratio (OR) = 3.42, 95% confidence interval (CI) = 2.86-4.09). |
| Emami et al, 2020 | literature review | 76993 | n/a | According to the meta-analysis, the pooled prevalence of hypertension, cardiovascular disease were estimated as 16.37% (95%CI: 10.15%-23.65%) and 12.11% (95%CI 4.40%-22.75%), respectively. |
| Liu et al, 2020 | literature review | 10,948 | n/a | Covid-19 patients who present with either, hypertension, CAD/CVD have a higher risk of developing severe disease, with an OR of 2.84 (95% CI 2.22 to 3.63) and 4.18 (95% CI 2.87 to 6.09), respectively. |
| Zali et al, 2020 | multi center cross sectional study | 16035 | Iran | The highest CFR was reported for patients who had diabetes and cardiovascular diseases (38.46%). |
| Gutierrez et al, 2020 | national reporting data analysis | 1,378,002 | Mexico | We found that obesity, diabetes, and hypertension are positively associated with the three outcomes in a synergistic manner. |
| Bajgain et al, 2020 | literature review | 22753 | n/a | Major comorbidities seen in overall population were CVD (8.9%), HTN (27.4%). |
| Hessami et al, 2020 | literature review | 159,698 | n/a | Results of meta-analysis indicated that acute cardiac injury, (OR: 13.29, 95% CI 7.35-24.03), hypertension (OR: 2.60, 95% CI 2.11-3.19), heart Failure (OR: 6.72, 95% CI 3.34-13.52), arrhythmia (OR: 2.75, 95% CI 1.43-5.25), coronary artery disease (OR: 3.78, 95% CI 2.42-5.90), and cardiovascular disease (OR: 2.61, 95% CI 1.89-3.62) were significantly associated with mortality. |
| Flaherty et al, 2020 | literature review | 17845 | n/a | Patients with pre-existing cardiovascular disease, especially hypertension and coronary heart disease, are at greatly increased risk of developing severe and fatal Covid-19 disease. |
| Wang et al, 2020 | literature review | 6263 | n/a | Specifically, pre-existing chronic conditions such as hypertension, cardiovascular diseaseare strongly associated with an increased risk of developing severe Covid-19. |

Table A3. Covid-19, COPD and other chronic respiratory illnesses: overview of the papers included in this literature review

| Author, year | Study type | Number of patients | Location | Main findings |
| --- | --- | --- | --- | --- |
| Natchtigall, 2020 | retrospective cohort study | 1904 | Germany | Pre-existing lung disease was one of the main predictors of death (HR 1.61; 95%CI 1.20 - 2.16) |
| Graziani et al, 2020 | clinical analysis of electronic records | 793 | Spain | Compared with COPD-free individuals, COPD patients with Covid-19 showed significantly poorer disease prognosis, as evaluated by hospitalizations (31.1% vs. 39.8%: OR 1.57; 95% CI 1.14–1.18) and mortality (3.4% vs. 9.3%: OR 2.93; 95% CI 2.27–3.79). |
| Lu et al, 2020 | literature review | 16095 | n/a | Chronic lung disease (OR = 3.43, 95% CI 1.80–6.52) was one of the key mortality risk factors. |
| Giannouchos et al, 2020 | retrospective case series analysis | 236,439 | Mexico | COPD was associated with hospitalization and adverse outcome. |
| Awortwe et al, 2020 | literature review | 5586 | n/a | Results indicated that chronic obstructive pulmonary disease, inter alia, worsen the clinical outcomes including mortality (risk difference RD 0.12, 95 %-CI 0.05􀀀 0.19, p =0.001), admission to ICU (RD 0.10, 95 %-CI 0.04􀀀 0.16, p = 0.001) and severe infection (RD 0.05, 95 %-CI 0.01􀀀 0.09, p = 0.01) in Covid-19 patients. |
| Noor et al, 2020 | literature review | 122,191 | n/a | In addition, significant association for high risk of mortality were also found for cerebrovascular disease, COPD, coronary heart disease, chronic renal disease, chronic liver disease, chronic lung disease and chronic kidney disease. |
| Mahumud et al, 2020 | literature review | 202,005 | n/a | The most prevalent chronic comorbid conditions were, inter alia, respiratory diseases (5%; 95% CI 3–6%). |
| Sepandi et al, 2020 | literature review | 944 | n/a | Comorbidity with some chronic diseases such Respiratory disorder 3.09 (1.39-6.88) can increase the risk of Covid19 mortality. |
| Khan et al, 2020 | literature review | 27,670 | n/a | The higher likelihood of deaths was found among Covid-19 patients who had pre-existing respiratory diseases (OR = 1.94, 95% CI = 1.72-2.19). |
| Liu et al, 2020 | literature review | 10,948 | n/a | Covid-19 patients who present with, inter alia, chronic pulmonary disease have a higher risk of developing severe disease, with an OR of 3.83 (95% CI 2.15 to 6.80). |
| Bajgain et al, 2020 | literature review | 22753 | n/a | Major comorbidities seen in overall population was, inter alia, COPD (7.5%). |

Table A4. Covid-19 and chronic kidney disease: overview of the papers included in this literature review

| Author, year | Study type | Number of patients | Location | Main findings |
| --- | --- | --- | --- | --- |
| Giannouchos et al, 2020 | retrospective case series analysis | 236,439 | Mexico | Chronic renal disease was associated with hospitalization and adverse outcome |
| Awortwe et al, 2020 | literature review | 5586 | n/a | Results suggested that chronic kidney disease, inter alia, was associated with worsen the clinical outcomes including mortality (risk difference RD 0.12, 95 %-CI 0.05􀀀 0.19, p =0.001), admission to ICU (RD 0.10, 95 %-CI 0.04􀀀 0.16, p = 0.001) and severe infection (RD 0.05, 95 %-CI 0.01􀀀 0.09, p = 0.01) in Covid-19 patients. |
| Noor et al, 2020 | literature review | n/a | n/a | In addition, significant association for high risk of mortality were also found for cerebrovascular disease, COPD, coronary heart disease, chronic renal disease, chronic liver disease, chronic lung disease and chronic kidney disease. |
| Sepandi et al, 2020 | literature review | n/a | n/a | The Comorbidity with some chronic diseases such as Kidney disorder OR: 2.61(1.22-5.60) can increase the risk of Covid19 mortality. |
| Ssentonoga et al, 2020 | literature review | n/a | n/a | chronic kidney disease (3.25 [1.13 to 9.28)], n = 9) was associated with a significantly greater risk of mortality from Covid-19. |
| Khan et al, 2020 | literature review | n/a | n/a | The higher likelihood of deaths was found among Covid-19 patients who had pre-existing renal (OR = 3.02, 95% CI = 2.60-3.51). |
| Bajgain et al, 2020 | literature review | 22753 | n/a | Major comorbidities seen in overall population were, inter alia, CKD (2.6%). |
| Gagliardi et al, 2020 | literature review | n/a | n/a | In this article, attention is focused on the epidemiology, etiology and pathophysiological mechanisms of kidney damage, histopathology, clinical features in nephropathic patients (CKD, hemodialysis, peritoneal dialysis, AKI, transplantation). |
| Wang et al, 2020 | literature review | 6263 | n/a | Specifically, pre-existing chronic conditions such chronic kidney disease are strongly associated with an increased risk of developing severe Covid-19. |

Table A5. Covid-19 and cancer: overview of the papers included in this literature review

| Author, year | Study type | Number of patients | Location | Main findings |
| --- | --- | --- | --- | --- |
| Zhang et al, 2020 | retrospective cohort study | 28 | Wuhan, China | A total of 15 (53.6%) patients had severe events and the mortality rate was 28.6%. If the last antitumour treatment was within 14 days, it significantly increased the risk of developing severe events [hazard ratio (HR) 4.079, 95% confidence interval (CI) 1.08 - 15.322, P=0.037]. |
| Noor et al, 2020 | literature review | 122,191 | n/a | An significant association were found between mortality among Covid-19 infected patients and cancer [RR 2.31, 95% CI (1.80–2.97), p < 0.001]. |
| Mahumud et al, 2020 | literature review | 202,005 | n/a | The most prevalent chronic comorbid conditions were, inter alia, other chronic diseases (e.g., cancer) (8%; 95% CI 6–10%). |
| Hu et al, 2020 | literature review | 47,344 | n/a | The prevalence of malignancy aong Covid-19 patients was 1.2 % (95CI 0.5–1.8 %). |
| Ssentonoga et al, 2020 | literature review | 65,484 | n/a | Cancer (1.47 [1.01 to 2.14), n = 10) was associated with a significantly greater risk of mortality from Covid-19. |
| Khan et al, 2020 | literature review | 27,670 | n/a | Higher likelihood of deaths was found among Covid-19 patients who had any types of cancers (OR = 2.22, 95% CI = 1.63-3.03). |
| Zali et al, 2020 | multi center cross sectional study | 16035 | Iran | The highest CFR was reported for patients who had, inter alia, cancer (35.79%). |
| Bajgain et al, 2020 | literature review | 22753 | n/a | Major comorbidities seen in overall population were, inter alia, Cancer (3.5%). |
| Zhang et al, 2021* | Literature review | 3019 | n/a | The evidence from the literature review suggests that there is a higher mortality/fatality from Covid-19 among cancer patients. |
| Abdul-Jawad et al, 2021* | Comparative study between cancer and non-cancer patients with Covid-19 | 41 cancer patients and 35 controls | UK | Heterogeneity of impact of Covid-19 on cancer patients, with solid cancer patients being more comparable to the general population, unlike patients with haematological cancers. |

- Added at a request of one of the reviewers

Table A6. Covid-19 and chronic liver disease: overview of the papers included in this literature review

| Author, year | Study type | Number of patients | Location | Main findings |
| --- | --- | --- | --- | --- |
| Oyelade et al, 2020 | literature review | 5595 | USA | In patients with Covid-19 and underlying liver diseases, 57.33% (43/75) of cases were severe, with 17.65% mortality |
| Noor et al, 2020 | literature review | 122,191 | n/a | In addition, significant association for high risk of mortality were also found for cerebrovascular disease, COPD, coronary heart disease, chronic renal disease, chronic liver disease, chronic lung disease and chronic kidney disease. |
| Khan et al, 2020 | literature review | 27,670 | n/a | The higher likelihood of deaths was found among Covid-19 patients who had pre-existing liver diseases (OR = 2.35, 95% CI = 1.50-3.69). |
| Wang et al, 2020 | literature review | 6263 | n/a | surprisingly, however, we found no correlation between chronic liver disease and increased disease severity. |

Table A7. Covid-19 and asthma: overview of the papers included in this literature review

| Author, year | Study type | Number of patients | Location | Main findings |
| --- | --- | --- | --- | --- |
| Chibba et al, 2020 | demographic and clinical feature analysis of patients with asthma | 1526 | USA | Asthma was not associated with an increased risk of hospitalization (relative risk, 0.96; 95% CI, 0.77-1.19) after adjusting for age, sex, and comorbidities. |
| Morais-Almeida et al, 2020 | literature review | n/a | n/a | There is no strong evidence supporting that patients with asthma have a higher risk of becoming seriously ill from coronavirus disease 2019 (COVID- 19). |
| Choi et al, 2021* | Study of linked medical claims data | 7590 | South Korea | 2.9% of Covid-19 patients had asthma. However, asthma was not considered as an independent risk factor for the clinical outcomes of Covid-19, after adjusting for correlates. |
| Terry et al, 2021* | Literature review | n/a | n/a | The results of the literature review do not provide clear evidence of increased risk of COVID-19 diagnosis, hospitalization, severity, or mortality due to asthma. |
| Sunjaya et al, 2021* | Literature review and meta-analysis | n/a | n/a | The prevalence of asthma among those infected with COVID-19 was 7.46%. There was no significant difference in the combined risk of requiring admission to ICU and/or receiving mechanical ventilation for people with asthma |
| Hussein et al, 2021* | Literature review and meta-analysis | n/a | n/a | There was no differential risk of hospitalization rate,  ICU admission, or development of acute respiratory distress syndrome (ARDS) between asthmatic and non-asthmatic cohorts. |
| Mendes et al, 2021* | Literature review | n/a | n/a | On average, asthma prevalence among Covid-19 patients is 1.6% and slightly lower than the global average. |

- Added at the request of a reviewer
